# Intravenous lidocaine as an adjuvant to propofol sedation for elderly patients undergoing same-visit bidirectional endoscopy: protocol for a multicenter randomized controlled trial

**DOI:** 10.1080/07853890.2025.2548389

**Published:** 2025-08-20

**Authors:** Ying Liu, Nan Song, Jing-tao Zhang, Jing Yan, Ke Peng, Hong Liu, Xi-sheng Shan, Fu-hai Ji

**Affiliations:** ^a^ Department of Anesthesiology, First Affiliated Hospital of Soochow University, Suzhou, China; ^b^ Institute of Anesthesiology, Soochow University, Suzhou, China; ^c^ Department of Anesthesiology, Zhangjiagang Second People’s Hospital, Suzhou, China; ^d^ Department of Anesthesiology and Pain Medicine, University of California Davis Health, Sacramento, California, USA

**Keywords:** Lidocaine, propofol, bidirectional endoscopy, desaturation, hypotension

## Abstract

**Background:**

Same-visit bidirectional endoscopic procedures under sedation are frequently performed in elderly patients. However, the optimal sedation regimen for elderly patients remains uncertain. This study seeks to evaluate the hypothesis that intravenous lidocaine, when used as an adjunct to propofol sedation, reduces the incidence of sedation-related adverse events during these procedures.

**Methods:**

This multicenter, randomized, double-blind, placebo-controlled trial will enroll 648 elderly patients scheduled for same-visit bidirectional endoscopy under sedation at four hospitals in China. Participants will be randomized in a 1:1 ratio to receive either intravenous lidocaine or normal saline (placebo), stratified by study center (*n* = 324 per group). All patients will receive sufentanil (0.1 μg/kg) followed by either lidocaine (1.5 mg/kg) or an equal volume of normal saline, and then propofol (1.0 mg/kg) for induction. Propofol will be titrated to maintain the target sedation level. The primary outcome is a composite of desaturation (peripheral oxygen saturation < 90%) and hypotension (systolic blood pressure < 90 mmHg or > 20% reduction from baseline). Secondary outcomes include total propofol dose, incidence of involuntary body movements, postoperative pain and fatigue scores, and recovery time. Analyses will follow a modified intention-to-treat approach.

**Discussion:**

We hypothesize that adjunctive lidocaine with propofol-based sedation will reduce the incidence of intraoperative desaturation and hypotension in elderly patients undergoing same-visit bidirectional endoscopy. The findings will contribute to optimizing sedation strategies in this vulnerable population.

**Trial registration:**

Chinese Clinical Trial Registry (ChiCTR2400087583)

## Introduction

The incidence of gastrointestinal diseases increases with advancing age, and endoscopic procedures are frequently performed in elderly patients for both diagnosis and therapeutic purposes [1–3]. To improve procedural efficiency and quality of care, an increasing number of elderly individuals opt to undergo esophagogastroduodenoscopy and colonoscopy during the same hospital visit [4]. Sedation is commonly employed during these procedures to enhance patient comfort and procedural satisfaction [5]. However, age-related physiological changes such as reduced drug metabolism and increased sensitivity to sedative agents heighten the risk of sedation-related complications in elderly patients [3]. Accordingly, minimizing sedative exposure is essential for enhancing the safety profile of gastrointestinal endoscopy in this population.

Propofol remains the most widely used agent for intravenous anesthesia in clinical practice due to its rapid onset, short duration of action, and favorable recovery profile [6,7]. Nevertheless, it is associated with dose-dependent adverse effects, most notably hypotension and respiratory depression [8,9]. These complications are particularly concerning in elderly patients and may adversely affect both intraoperative stability and postoperative recovery [9,10]. Intravenous lidocaine has emerged as a promising adjunct to sedation protocols. Prior studies have demonstrated that intravenous lidocaine reduces propofol requirements and alleviates post-colonoscopy visceral pain [11]. Moreover, recent evidence suggests that intravenous lidocaine may significantly improve sedation quality and endoscopist satisfaction, and even reduce the incidence of sedation-related adverse events during endoscopic retrograde cholangiopancreatography (ERCP) procedures [12–14]. It remains uncertain whether the adjunctive use of lidocaine during same-visit bidirectional endoscopy can reduce the incidence of sedation-related adverse events in elderly patients.

This multicenter, randomized, double-blind, placebo-controlled trial is designed to test the hypothesis that lidocaine, administered as an adjunct to propofol sedation, lowers the incidence of desaturation and hypotension during same-visit bidirectional endoscopy in elderly patients.

## Methods

### Ethic approval and trial registration

The study protocol was approved by the ethics committees of the leading center (the First Affiliated Hospital of Soochow University, Suzhou; Approval No. 2024–028) and all participating centers. This trial was registered with the Chinese Clinical Trial Registry (http://www.chictr.org.cn; identifier: ChiCTR2400087583) prior to patient enrollment.

### Study design

**Figure 1.**
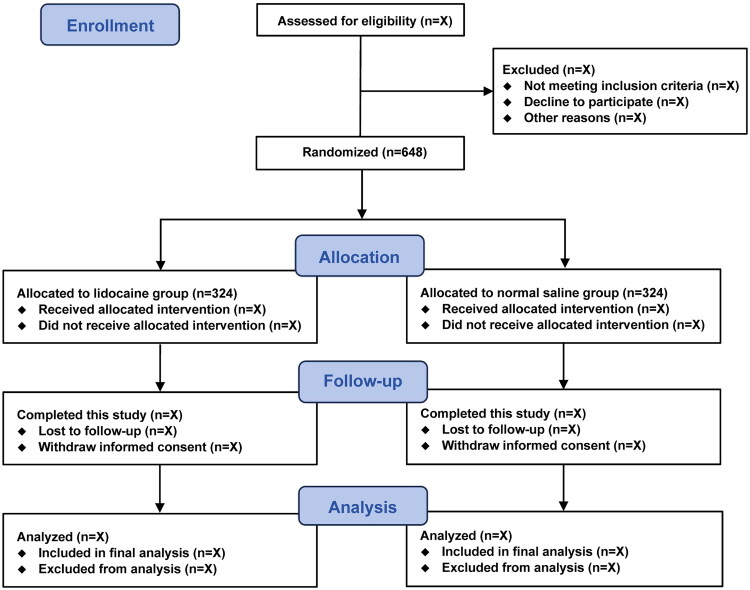
Study flowchart.

This investigator-initiated, multicenter, randomized, double-blind, placebo-controlled trial is being conducted at four hospitals in China. This trial commenced on August 2024, and is expected to conclude on December 2025. Eligible patients must provide written informed consent prior to enrollment. A total of 648 eligible patients undergoing same-visit bidirectional endoscopy will be randomly assigned in a 1:1 ratio to receive either lidocaine or normal saline (324 patients per group). The study flowchart is presented in Figure 1, while Table 1 outlines the schedule for participant enrollment, study interventions, and outcome assessments in accordance with the SPIRIT statement (Supplemental file).

**Table 1.** Schedule of patient enrollment, study interventions, and outcome measurements.

	Study period					
	Enrollment	Allocation	Post-Allocation			Close-Out
	Anesthesia Clinic Visit	Prior to Sedation	During Sedation	Emergence from Sedation	15 Min after Procedure	Hospital Discharge
**Enrollment**						
Eligibility screening	**×**					
Written informed consent	**×**					
Demographic data	**×**					
Baseline characteristics	**×**					
Randomization		**×**				
Allocation		**×**				
**Interventions**						
Lidocaine			**×**			
Normal saline placebo			**×**			
**Measurements**						
Desaturation events			**×**			
Hypotension events			**×**			
Total dose of propofol			**×**			
Involuntary body movement			**×**			
VAS pain scores					**×**	
VAS fatigue scores					**×**	
Recovery time				**×**	**×**	
Injection pain				**×**	**×**	
Dizziness				**×**	**×**	
Headache				**×**	**×**	
Nausea and vomiting				**×**	**×**	
Endoscopist satisfaction			**×**			
Patient satisfaction					**×**	
Sleep score (RCSQ)						**×**
Appetite score (AFSQ)						**×**

According to SPIRIT statement of defining standard protocol items for clinical trials.

Abbreviation: RCSQ: Richards-Campbell Sleep Questionnaire; AFSQ: Appetite and Food Satisfaction Questionnaire

### Eligibility criteria

Eligible participants were (1) aged between 65 and 80 years, (2) American Society of Anesthesiologists (ASA) physical status classification of I to Ш, (3) body mass index (BMI) between 18 and 30 kg/m^2^, (4) scheduled to undergo same-visit bidirectional endoscopy under sedation.

Exclusion criteria were as follows: (1) contraindications to endoscopic procedures or refusal of anesthesia, (2) uncontrolled or potentially life-threatening cardiovascular and respiratory conditions, including acute coronary syndrome, severe uncontrolled hypertension (systolic blood pressure ≥ 180 mmHg or diastolic blood pressure ≥ 110 mmHg), serious arrhythmias, heart failure, acute respiratory infections, asthma exacerbations, active hemoptysis, severe hepatic dysfunction (Child-Pugh class C or higher), acute upper gastrointestinal bleeding with shock, severe anemia (hemoglobin < 6 g/dL), or gastrointestinal obstruction with gastric content retention, (3) absence of an accompanying escort or guardian, (4) known allergies to sedatives or anesthetics, or a significant personal or family history of allergic reactions.

### Randomization and blinding

An independent statistician, who is not involved in the study, will generate the randomization list using an online tool (https://www.sealedenvelope.com/simple-randomiser/v1/lists). Participants will be randomized in a 1:1 ratio using permuted block sizes of 2 and 4, stratified by study center. The randomization assignments will be prepared and sealed in sequentially numbered, opaque envelopes by an independent research assistant who is not engaged in participant recruitment, intervention administration, or outcome assessment. These envelopes will be securely stored until allocation. An independent researcher, responsible for patient enrollment, will open the corresponding envelope to obtain the randomization assignment and prepare the sedation medications accordingly. All patients will receive either lidocaine or normal saline according to the randomization schedule. As both lidocaine and normal saline are clear and colorless solutions, the contents of the syringes will be visually indistinguishable. To minimize the risk of unblinding associated with lidocaine administration, a low dose of sufentanil will be administered at the initiation of anesthesia induction, and intravenous access will be established *via* the antecubital vein [15]. Patients, peri-procedure anesthesiologists, gastroenterologists, and outcome assessors remain blinded to the assigned treatment groups.

### Perioperative management and study interventions

The patient’s vital signs, including heart rate, peripheral oxygen saturation (SpO_2_), and noninvasive blood pressure (measured at 2-minute intervals), are continuously monitored throughout the procedure. All patients will receive oxygen *via* a nasal cannula at a flow rate of 3 L/min for the duration of examination. Gastrointestinal endoscopy is performed sequentially by a team of skilled gastroenterologists at each study center, beginning with gastroscopy followed by colonoscopy. Upon completion of the procedure, patients were transferred to the post-anesthesia care unit (PACU) for postoperative monitoring.

During anesthesia procedures, all agents will be administered at the slowest feasible rate to optimize patient safety and pharmacologic efficacy. Patients in the lidocaine group will receive sufentanil 0.1 µg/kg, lidocaine 1.5 mg/kg, and propofol 1.0 mg/kg administered sequentially at induction. Patients in the saline group will receive an equal volume of normal saline in place of lidocaine, with all other procedures identical to the lidocaine group. Sedation will be titrated by adjusting the propofol dose (typically 0.2–0.3 mg/kg) throughout the procedure to achieve the predetermined target level of sedation. The depth of sedation will be assessed every 30 s using Modified Observer’s Alertness/Sedation scale (MOAA/S) score [16]. At the initiation of gastroscopy, the target sedation depth was set at a MOAA/S score of 1 (only in response to a compressive trapezius stimulus). For the ensuing colonoscopy, the target sedation depth will be adjusted to a MOAA/S of 2 (responding only to light prodding or shaking) [7,17].

Baseline blood pressure will be determined as the average of two measurements obtained while the patient is seated calmly in the preoperative waiting area. Hypotension, defined as a systolic blood pressure (SBP) < 90 mmHg or a reduction of more than 20% from baseline, will be managed with intravenous administration of ephedrine (5–10 mg) or phenylephrine (50–100 μg), as clinically indicated. Bradycardia, defined as a heart rate < 45 beats per minute, will be treated with intravenous atropine (0.3–0.5 mg), or ephedrine bolus if accompanied by hypotension. In the event of hypoxemia (SpO_2_ < 90%), initial management will include a jaw thrust maneuver, increasing oxygen flow to 5–10 L/min, and providing mask-assisted positive pressure ventilation, as necessary. If adequate ventilation cannot be maintained, emergency placement of a laryngeal mask airway or endotracheal intubation will be considered. All adverse events and corresponding interventions will be promptly addressed and thoroughly documented.

### Data collection

Demographic data include age, sex, weight, height, and BMI. Baseline characteristics comprise smoking status, medication history, comorbidities, and ASA physical status. Other peri-procedure variables encompass the total propofol dose, duration of endoscopy, and time to emergence following procedure completion. Hemodynamic parameters, including blood pressure, heart rate, and peripheral SpO_2,_ will be recorded at baseline, after induction, 1 min after procedure initiation, at the end of procedure, and upon emergence. All data will be recorded in electronic case report forms (eCRFs) under the supervision of qualified research personnel. An electronic trial database will be constructed from these eCRFs. Upon completion of data entry, the database will be de-identified and locked. The dataset will then be transferred to an independent statistician for final analysis in accordance with the prespecified statistical analysis plan.

An independent Data Monitoring Committee (DMC) has been established to oversee data integrity and address any issues arising during data collection and registration. The DMC comprises a chair (an experienced anesthesiologist), a pharmacologist, a gastroenterologist and a professor of statistics. In the event of uncertainties or discrepancies related to data collection or registration, the DMC will convene to discuss and reach a consensus resolution.

### Study outcomes

The primary outcome is a composite of desaturation and hypotension events occurring during the procedure [7,17]. Desaturation is defined as peripheral SpO_2_ < 90% for 10 s or longer, while hypotension is defined as a systolic blood pressure (SBP) < 90 mmHg or a reduction in SBP of more than 20% from baseline. The composite endpoint was considered met if either respiratory depression or hypotension occurs in a participant.

The secondary outcomes include (1) total dose of propofol administered, (2) incidence of involuntary body movement during the procedure; (3) postoperative pain score at 15 min after the procedure, assessed using Visual Analog Scale (VAS, 0–10; 0 = no pain, 10 = the most severe pain); (4) fatigue score at 15 min after the procedure, assessed using VAS (0–10; 0 = no fatigue, 10 = extreme fatigue); (5) recovery time, defined as the interval from the end of surgical examination to the point when the patient is able to respond accurately to simple questions (e.g. basic arithmetic). Minimal clinically important differences (MCID) were applied to interpret VAS pain and fatigue scores, defined as a 1-point change on the 0–10 VAS scale for both measures [18,19].

Other outcomes include (1) incidence of other adverse events: injection pain, dizziness, headache, nausea and vomiting; (2) endoscopist satisfaction, assessed on a 5-point scale (0–5; 0 = very dissatisfied, 5 = highly satisfied); (3) patient satisfaction (0–5; 0 = very dissatisfied, 5 = highly satisfied); (4) sleep score on the night following the procedure, assessed using Richards-Campbell Sleep Questionnaire (RCSQ) [20]; (5) appetite score on the first postoperative day, assessed using the Appetite and Food Satisfaction Questionnaire (AFSQ) [21]. Sleep and appetite assessments will be conducted *via* telephone between 7 am and 9 am on the day following the procedure.

### Sample size calculation

In our prospective study conducted from January 2023 to June 2023, we enrolled 50 eligible patients undergoing same-visit bidirectional endoscopy under propofol-based sedation. The results indicated that 8 patients (16%) experienced desaturation and 7 patients (14%) developed hypotension (unpublished data). These incidences are consistent with the recent literature [22]. Prior studies have demonstrated that the addition of lidocaine to propofol for painless gastroenteroscopy or retrograde cholangiopancreatography can reduce the incidence of respiratory depression and hypotension by approximately 8 to 30% [12,13]. Based on these findings, we hypothesize that the addition of lidocaine will reduce the incidence of respiratory depression and hypotension by 10%, lowering their combined occurrence to 20%. Assuming a two-sided significance level (α) of 0.05 and a power (1-β) of 80%, a total of 290 patients per group will be required. Accounting for an anticipated dropout rate of 10%, we plan to recruit a total of 648 patients, with 324 allocated to each group. Sample size calculations were performed using the PASS software (Version 15, PASS Institute Inc.).

### Statistical analysis

Continuous variables will be assessed for normality using the Shapiro–Wilk test and will be presented as mean ± standard deviation (SD) for normally distributed data, or as median with interquartile range (IQR) for non-normally distributed data. Between-group comparisons will be conducted using the independent-samples t-test or the Mann–Whitney U test, depending on the distribution. Categorical variables will be reported as counts (percentages) and compared using the chi-squared test or Fisher’s exact test, as applicable.

Demographic and baseline characteristics will be summarized using descriptive statistics and standardized mean differences will be reported to assess the balance between groups. For study outcomes, the treatment effects between the two groups will be evaluated using odds ratios (OR) or mean differences (MD), each accompanied by 95% confidence intervals (CI). Additionally, outcomes will be further analyzed using multivariate logistic regression or generalized linear models, adjusting for relevant baseline covariates (age, BMI, history of hypertension, chronic obstructive pulmonary disease [COPD], and diabetes mellitus) and study center. Subgroup analyses of the primary outcome will be conducted to explore potential differential effects of the intervention across predefined subgroups, including BMI (< 25 kg/m^2^ vs ≥ 25 kg/m^2^), history of hypertension (yes vs no), history of COPD (yes vs no), history of diabetes mellitus (yes vs no), and study center (leading center vs participating center), with interaction effects evaluated using logistic regression. Sensitivity analyses will evaluate individual components of the primary outcome and the sedation-related adverse events (desaturation, hypotension, dizziness, headache, nausea and vomiting). Correction for multiple comparisons among secondary outcomes will be performed using the Benjamini–Hochberg method to control the false discovery rate.

All analyses will follow the modified intention-to-treat principle, including all randomized patients who undergo same-visit bidirectional endoscopy under sedation and have available primary outcome data. Protocol deviations are expected to be minimal. No interim analyses or imputation for missing data are planned. All statistical tests will be two-sided, with a P value < 0.05 considered statistically significant. Statistical analyses will be performed using R software (version 4.3.0; R Foundation for Statistical Computing).

## Discussion

This multicenter, randomized, double-blind, parallel-controlled trial will include 648 eligible patients to evaluate the effects of intravenous lidocaine versus normal saline placebo on the incidence of desaturation and hypotension during same-visit bidirectional endoscopy. Furthermore, we will assess the total propofol consumption, incidence of involuntary body movements during procedure, postoperative pain and fatigue levels, recovery time, injection pain, dizziness, headache, nausea and vomiting, satisfaction of endoscopists and patients, as well as postoperative sleep quality and appetite. The primary hypothesis is that lidocaine, when used as an adjunct to propofol sedation, will reduce the incidence of intraoperative desaturation and hypotension in elderly patients undergoing same-visit bidirectional endoscopy. This trial will be conducted and reported in accordance with the Consolidated Standards of Reporting Trials guidelines.

As a preferred sedative for gastrointestinal endoscopy, propofol offers advantages over conventional agents like benzodiazepines, including faster induction, quicker recovery, and enhanced patient satisfaction [23]. However, its administration is associated with a relatively high risk of cardiorespiratory adverse events. Reported complication rates vary considerably, ranging from 20% to 60%, depending on the procedure type and the sedation strategy employed [8,11,22,24]. In elderly patients, common age-related physiological changes, such as reduced hepatic and renal drug clearance, heightened sensitivity to sedative agents, impaired protective airway reflex and increased secretions production during procedures, further increase the risk of sedation related complications [2,25]. According to the 2006 guidelines on sedation for gastrointestinal endoscopy, over-sedation remains a major contributor to adverse events, and reduced sedative dosages are recommended in older populations [26].

Lidocaine is a cost-effective, commonly used local anesthetic with well-established antinociceptive and anti-inflammatory properties [27]. Intravenous lidocaine administration has been shown to reduce intraoperative volatile anesthetics by approximately 30–40%, as well as decrease propofol requirements during total intravenous anesthesia [28,29]. During endoscopic procedures, patient discomfort primarily arises from visceral nociception induced by distension and traction of the gastrointestinal tract. The sedative-sparing effect of intravenous lidocaine has also been demonstrated in endoscopic settings. In a randomized, placebo-controlled trial involving 40 patients undergoing colonoscopy, adjunctive intravenous lidocaine infusion reduced propofol consumption by 50% (mean dose: 58 mg vs 121 mg) and significantly improved postoperative pain and fatigue levels [11]. In addition to the propofol-sparing effect, lidocaine infusion has been shown to enhance the ventilatory response to carbon dioxide in humans [30]. Furthermore, Intravenous lidocaine at doses of 1–2 mg/kg is effective in preventing laryngospasm during general anesthesia [31]. Collectively, these effects may contribute to a reduction in the incidence of sedation-related adverse events. Several patient- and disease-related factors, including age, BMI, history of hypertension, COPD, and diabetes mellitus, are considered as potential confounders for sedation-related adverse events [32]. These variables will be adjusted for using multivariate logistic regression or generalized linear models to minimize bias and more precisely estimate the independent effect of the intervention on the study outcomes.

In elderly individuals, a significant increase in the apparent volume of distribution of lidocaine has been observed. They also exhibit a markedly prolonged elimination half-life compared with younger adults (2.7 vs. 1.6 h) [33]. Although hepatic blood flow is reduced in the elderly, previous studies have shown no significant change in the plasma metabolic clearance of lidocaine in this population [34]. Consequently, the recommended initial loading dose is the same as that for younger adults, whereas the maintenance infusion should be reduced to mitigate the risk of drug accumulation [33]. In the general population, the pharmacological effects of a single intravenous bolus of lidocaine typically persist for 10–20 min [27]. In our previous study involving patients undergoing same-visit bidirectional endoscopy, the median procedure time was 18–20 min [7]. Therefore, in the present protocol, we adopted a loading dose of 1.5 mg/kg, consistent with the regimen described by Forster et al. [11], without the use of continuous infusion. To our knowledge, this will be the first multicenter randomized controlled trial to evaluate whether adjunctive lidocaine administration can reduce the incidence of cardiorespiratory complications associated with propofol-based sedation in elderly patients undergoing same-visit bidirectional endoscopy.

This study has several limitations. First, intravenous lidocaine may elicit a metallic taste, potentially introducing subtle unblinding cues. Second, adjunctive lidocaine is administered prior to propofol to explore a potentially more suitable sedation strategy for elderly patients, however, this sequence may also introduce the risk of unblinding. To mitigate this, a low dose of sufentanil is administered before lidocaine or placebo, and intravenous access is established *via* the antecubital vein in our study to minimize sensory cue. Third, patients older than 80 years are excluded from this study, as we believe that the optimal sedation regimen for the very elderly patients should be determined based on the findings of the current study.

In conclusion, this multicenter randomized controlled trial will assess the impact of intravenous lidocaine as an adjuvant to propofol-based sedation on the incidence of desaturation and hypotension in elderly patients undergoing same-visit bidirectional endoscopy. Regardless of the outcome, the findings of this study will provide valuable evidence to guide and optimize sedation practices in this vulnerable population.

## Supplementary Material

SPIRIT checklist.doc

## Data Availability

Data sharing is not applicable to this article as no data were created or analysed in this study.
